# The VEST External Support for Saphenous Vein Grafts in Coronary Surgery: A Review of Randomized Clinical Trials

**DOI:** 10.3390/jcdd10110453

**Published:** 2023-11-07

**Authors:** Giovanni Jr. Soletti, Michele Dell’Aquila, Lamia Harik, Gianmarco Cancelli, Talal Alzghari, Roberto Perezgrovas-Olaria, Arnaldo Dimagli, Kevin R. An, Jordan Leith, Camilla Sofia Rossi, Christopher F. Barile, Michelle Demetres, Christopher Lau, Leonard N. Girardi, Mario Gaudino

**Affiliations:** 1 Department of Cardiothoracic Surgery, Weill Cornell Medicine, 525 East 68th Street, New York, NY 10021, USA; 2 Samuel J. Wood Library and C.V. Starr Biomedical Information Centre, Weill Cornell Medicine, New York, NY 10021, USA

**Keywords:** coronary artery bypass grafting (CABG), saphenous vein graft, intimal hyperplasia, occlusion, patency, VEST, device, external support

## Abstract

Saphenous vein grafts (SVGs) are the most frequently used conduits in coronary artery bypass grafting (CABG), but their higher rate of occlusion compared to arterial conduits remains a concern. Previous studies have shown that SVG failure is mainly driven by intimal hyperplasia, an adaptative response to higher pressures of the arterial circulation. The VEST^TM^ device (Vascular Graft Solutions, Tel Aviv, Israel), an external support designed to mitigate intimal hyperplasia in SVGs, has been tested in few clinical trials (RCTs). Herein, we descriptively evaluated the randomized evidence on the VEST device.

## 1. Introduction

Coronary artery bypass grafting (CABG) is the most commonly performed cardiac surgery procedure in the United States and worldwide [1]. Although there has been an increase in the adoption of multi-arterial grafting strategies over the previous decade, saphenous vein grafts (SVGs) continue to be, by far, the most frequently used conduits [1]. However, the main limitation to their use is a higher rate of occlusion compared to arterial conduits [2,3].

Previous studies have described the main processes leading to SVG failure namely thrombosis, intimal hyperplasia, and accelerated atherosclerosis. Thrombosis represents the main determinant of SVG failure within the first post-operative month, whereas intimal hyperplasia (an adaptative response to higher pressures of the arterial circulation) is the main mechanism of graft failure within the first year [4,5].

The VEST^TM^ device (Vascular Graft Solutions, Tel Aviv, Israel), an external support for SVGs, has been proposed as a mechanical approach to prevent intimal hyperplasia, minimizing SVG dilatation under pressure. By targeting post-implantation dilatation with its arterial biomechanical properties, it has been proposed this device may potentially improve graft flow patterns and the subsequent development of intimal hyperplasia.

When tested in a pre-clinical model, VEST was found to significantly reduce SVG intimal hyperplasia (*p* < 0.02) early after CABG [6]. This finding opened the doors to the conduction of randomized clinical trials (RCTs) in human subjects.

Herein, we reviewed the available randomized evidence on the topic.

## 2. Materials and Methods

This review follows the PRISMA (preferred reporting items for systematic reviews and meta-analyses) guidelines for transparent reporting. Ethical approval was waived as data were solely collected from publicly available literature.

A PICO model (population/intervention/comparison/outcome) was used to formulate the clinical question. A literature search on Ovid MEDLINE (1946 to present), Ovid Embase (1974 to present), and the Cochrane Library was then performed by a qualified medical librarian (M.D.). Search results were reviewed by the first and senior authors (G.J.S. and M.G.) and, upon agreement on the accuracy, duplicates were removed. The titles and abstracts were then screened by two independent authors (G.C. and C.F.B.). Disagreement was resolved by the first author (G.J.S.). Eligible studies were then thoroughly reviewed along with their reference lists. Studies were included if they were randomized control trials (RCTs) comparing VEST-supported SVGs to non-supported SVGs in patients undergoing isolated CABG for multi-vessel coronary artery disease. All non-randomized evidence was excluded. No language or publication date restrictions were applied.

**Figure 1:**
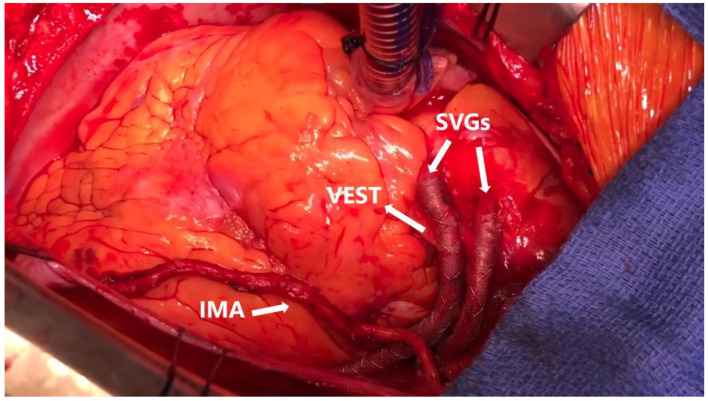
Intraoperative image showing the left internal mammary artery (left) and two VEST devices externally surrounding two saphenous vein grafts (right). IMA: internal mammary artery; SVG: saphenous vein graft. (Reprinted with permission from Sandner S, Angleitner P, Laufer G, Zimpfer D. External stent (VEST) for saphenous vein grafts in coronary artery bypass grafting. Multimed Man Cardiothorac Surg. 2019 Feb 1;2019. Doi: 10.1510/mmcts.2019.007. PMID: 30767438.)

The VEST^TM^ device (Vascular Graft Solutions, Tel Aviv, Israel) is an external cobalt–chrome support specifically designed for SVGs. A detailed description of how the device should be prepared and deployed has been previously published [7]. Briefly, after harvesting a vein graft without using metal clips to ligate its side branches, the distal anastomosis should be completed first. Graft characteristics should guide the selection of the appropriate stent, as VEST is available in two diameters and various lengths. Before completing the proximal anastomosis, the device should be slid over the vein graft using a cannula. By gently squeezing its distal end, the stent should be first extended to its maximum length and slid towards the distal anastomosis. With the same technique, while holding the device right below the un-extended segment, its proximal end should be stretched towards the proximal anastomosis, which can then be completed. The proximal and distal ends of VEST should be kept at a distance of 2 to 10 mm from the anastomoses in order to avoid any contact. After completion of the proximal anastomosis, the device should be adjusted to obtain the desired length and diameter (Figure 1). Since two models of the device were being produced at the time of our searches, a company delegate was contacted who confirmed that the model used in all the RCTs selected for this review was the VEST 1.0.

## 3. Results

### 3.1. Search Results

**Figure 2:**
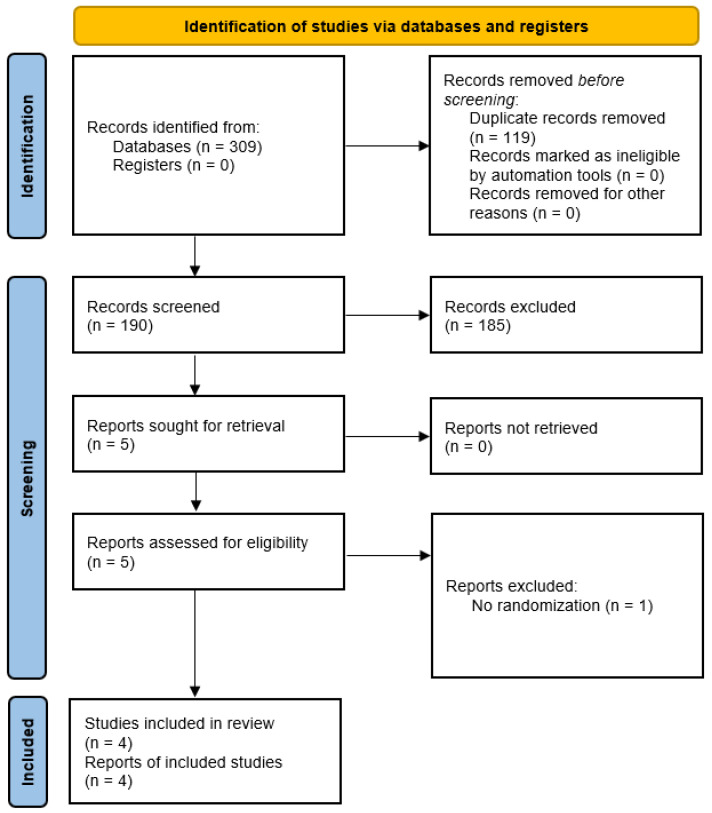
PRISMA flow diagram outlining the study selection process. PRISMA: preferred reporting items for systematic reviews and meta-analyses.

The PRISMA flow diagram outlining the study selection process is shown in Figure 2.

Our searches yielded 390 records. After deduplication, 190 were screened. Five studies and their reference lists were thoroughly reviewed. Four RCTs were included:

- VEST (Venous External Support Trial) I [8];
- VEST III [9];
- VEST IV (long-term follow-up of VEST I) [10];
- US VEST [11].

VEST II, though not an RCT, will be briefly described in relation to the lower patency rate observed in VEST-supported SVGs directed to the right coronary territory as opposed to those directed to the left territory in VEST I [12].

In addition, a key preclinical study, which allowed the progression to human RCTs, will also be concisely summarized [6].

### 3.2. Preclinical Model Study

The VEST device was tested for the first time in an ovine model of CABG [6]. The aim of the study was (1) to evaluate graft patency and quantification of luminal diameter using post-procedural and 12-week angiography; and (2) to assess for graft inflammation, injury, or thrombosis through histopathologic examination following the second angiography. Neointimal formation was also analyzed.

For this purpose, fourteen adult sheep underwent CABG using two vein grafts each, one to the left anterior descending artery and the other to the obtuse marginal artery.

Ten sheep survived. Three died perioperatively, but their death was deemed unrelated to the device implantation. While no difference in graft uniformity was found in post-procedural quantitative angiography between the groups, a significantly better uniformity was observed at the 12-week follow-up in VEST-supported vein grafts compared to non-supported vein grafts (*p* < 0.002).

Histologic examination showed no difference in terms of graft inflammation and injury. Among the surviving animals, three grafts were found to be thrombosed and one occluded, all in the control group (*p* = 0.043). Conversely, the mean neointimal area of supported grafts was found to be significantly lower than that of control grafts (11.2 mm^2^ versus 23.1 mm^2^; *p* < 0.02). Anastomotic sites appeared to be normal in both groups. The authors also noticed that the adventitial layer of supported grafts was incorporated into the device’s pores, but no device migration or damage were seen.

To summarize, this study demonstrated that the VEST device was able to reduce neo-intimal formation in vein grafts, holding the potential to improve long-term patency and paving the way for RCTs in humans.

### 3.3. The VEST Studies

#### 3.3.1. VEST I

Conducted between October 2011 and September 2012, VEST I was the pilot RCT to test the VEST device in human subjects [8], following successful results in the previously described preclinical model [6].

Its primary outcome compared the intimal hyperplasia area, as assessed via intravascular ultrasonography at 12 months between stented and non-stented SVGs. Secondary outcomes, assessed via angiography at 12 months, were SVG failure (>50% stenosis), ectasia (>50% initial diameter), and graft uniformity expressed as Fitzgibbon patency class.

To achieve this goal, 30 subjects with multi-vessel coronary artery disease scheduled for on-pump CABG, including a left internal thoracic artery to the left anterior descending coronary artery and SVGs to the right and circumflex territories, were enrolled. Each patient received one VEST to a single SVG to either the right or the circumflex territory, while one or more non-stented SVG served as control (within-patient study design).

Twenty-nine patients (29/30; 96.6%) completed the prespecified follow-up. At 12 months, 21 stented and 23 non-stented SVGs were analyzed via intravascular ultrasonography. Compared to non-stented SVGs, the intimal hyperplasia area of stented SVGs was reduced by 14.6% (*p* = 0.04). Although not significant, intimal-medial thickness was also found to be reduced by 11.9% (*p* = 0.06) in stented SVGs. A total of 30 stented and 39 non-stented SVGs were assessed via angiography at 12 months. Total graft failure did not significantly differ between the stented and non-stented groups (30% versus 28.2%; *p* = 0.55). Compared to non-stented SVGs, Fitzgibbon class I (perfect patency) and ectasia were, respectively, increased (*p* = 0.08) and reduced (*p* = 0.05) in stented SVGs.

Interestingly, the use of metallic clips to ligate SVG side branches, as opposed to sutures, was found to increase the SVG failure rate in the stented group. A possible explanation was that the optimal device–graft alignment was compromised by metal clips constrained within VEST, resulting in more deformation of the vessel wall. Since this higher failure rate was mainly observed in SVGs directed to the right territory, fixation of VEST to the proximal, distal, or both anastomoses was also hypothesized to be a contributing factor due to the acute angulation of SVGs on their course to the inferior wall.

In summary, this RCT suggested that the VEST device could reduce the intimal hyperplasia area one year after CABG, but an unexpectedly low patency in SVGs directed to the right coronary territory needed to be addressed.

#### 3.3.2. VEST II

VEST II was a prospective study specifically designed to address the lower patency of externally-stented SVGs to the right coronary territory (55–60%), as opposed to the left territory (85–90%), observed in VEST I [12]. As previously mentioned, this was due to (1) a surgical variation of the implantation technique consisting of device fixation to the proximal, distal, or both anastomoses using sutures; and (2) to the use of metal clips to ligate SVG side branches.

For this purpose, 30 patients received one VEST-supported SVG to the right coronary territory and one or more non-supported SVGs to the left territory. The primary outcome was SVG failure (occlusion or >50% stenosis) at 3–6 months as assessed via computerized tomography–angiography.

At follow-up, the patency of stented SVGs was 86.2%, while all non-stented SVGs to the left territory were patent. These results demonstrated that avoidance of fixation of VEST stents to the anastomoses improved SVG patency to the right coronary territory by 20–25%. Hence, this study was instrumental in reigniting interest in the use of VEST.

#### 3.3.3. VEST III

VEST III was a within-patient, multi-center RCT conducted in 14 European centers between 2015 and 2019 [9]. A total of 184 patients were enrolled and, in each of them, one SVG was randomized to an external stent and one non-stented SVG served as control. The primary outcome was graft patency expressed using the Fitzgibbon patency scale assessed via angiography, while the secondary outcome was intimal hyperplasia area at 2 years.

Patency rates were similar between stented and non-stented SVGs (78.3% versus 82.2%; *p* = 0.43). Compared to non-stented SVGs, VEST-SVGs were (1) more likely to show perfect patency (Fitzgibbon class I); and (2) less likely to show lumen irregularities involving more or less than 50% of grafts (Fitzgibbon class II-III) at the 2-year angiographic follow-up. VEST-SVGs also showed a significant reduction in intimal hyperplasia area (−22.5%; *p* < 0.001) and thickness (−23.5%; *p* < 0.001).

The rate of major adverse cardiac and cerebrovascular (MACCE) events at 2 years was 12%, with individual rates of all-cause mortality, stroke, and myocardial infarction being 2.7%, 3.3%, and 2.7%, respectively. Ischemia-driven reintervention was performed in 6% (11/183) of patients, of which 2.2% (4/183) were in the stented territory and 3.8% (7/183) were in the non-stented territory.

Taken together, these findings confirmed that the VEST device was able to reduce intimal hyperplasia area and medial-intimal thickness of SVGs, but this reduction did not improve graft patency rates or freedom from repeat revascularization.

#### 3.3.4. VEST IV

VEST IV analyzed the same cohort of patients enrolled in VEST I at a mean follow-up of 4.5 years [10]. However, follow-up data were collected in only 70% (21/30) of the initial population.

SVG failure rates were comparable between stented and non-stented grafts (30% versus 23%; *p* = 0.42). Fitzgibbon perfect patency (class I) remained significantly higher in stented versus non-stented SVGs (81% versus 48%; *p* = 0.002). Similarly, intimal hyperplasia area (4.27 ± 1.27 mm^2^ versus 5.23 ± 1.83 mm^2^; *p* < 0.001) and thickness (0.36 ± 0.09 mm versus 0.42 ± 0.11 mm; *p* < 0.001) were found to be significantly reduced in VEST-SVGs.

Follow-up mortality was 10% (3/30), with two deaths being cancer-related and one cardiac-related. Lastly, three revascularization events were observed: one to the non-stented territory, one to both the stented and non-stented territories, and one triggered by the protocol-mandated angiography control.

#### 3.3.5. US VEST

US VEST was a within-patient, multi-center RCT conducted between January 2018 and February 2019 [11]. A total of 224 patients were enrolled at 17 cardiothoracic surgical trials network centers in North America. For each patient, one SVG was randomized to an external support device, while another SVG was not supported and served as control. The primary outcome was intimal hyperplasia assessed via intravascular ultrasound at 12 months post-randomization, while the secondary outcomes were SVG lumen diameter uniformity assessed via angiography and expressed as Fitzgibbon patency classes, and SVG failure defined as 50% or greater graft stenosis via quantitative coronary angiography. MACCE were also collected through month 12.

Intravascular ultrasound at 12 months post-randomization revealed no statistically significant difference in the intimal hyperplasia area between supported and unsupported grafts (5.11 ± 0.16 mm^2^ versus 5.79 ± 0.20 mm^2^; *p* = 0.07).

Intimal-medial thickness was lower in the supported group (0.38 ± 0.14 mm versus 0.43 ± 0.16 mm; mixed model difference: −0.044, 95% CI: −0.075 to −0.013). No significant difference was observed in lumen diameter uniformity (0.13 ± 0.06 versus 0.13 ± 0.07; mixed model difference: 0.001, 95% CI: −0.011 to 0.014). Graft occlusion was observed in 47/203 (23.2%) supported grafts and 45/203 (22.2%) unsupported grafts.

It must be noted that the overall SVG occlusion rate in US VEST was 22.7%, nearly 75% higher than that anticipated in the protocol (13%) [11]. This discrepancy may be a direct consequence of the preferred use of the endoscopic harvesting technique (75.4%), which is known to increase SVG failure compared to the open technique [13]. In any case, this higher-than-expected occlusion rate led to the unusual circumstance wherein measurements of the primary outcome (intimal hyperplasia) were mostly derived via imputation methods, which was an important limitation.

The rate of MACCE at 12 months was 7.1%, with individual rates of all-cause mortality, stroke, and myocardial infarction being 2.2%, 2.7%, and 3.1%, respectively. The ischemic- and non-ischemic-driven target vessel revascularization of supported and unsupported grafts (or associated target coronary artery) were 1.8% and 2.2%, respectively.

In summary, this trial showed that the use of external scaffolding for SVGs did not result in a statistically significant reduction of intimal hyperplasia area compared to unsupported grafts (*p* = 0.07);however, these results were affected by imputation techniques.

A summary of the main characteristics and findings of the VEST studies is available in Table 1.

## 4. Discussion

In this review, we summarized the available randomized evidence regarding the VEST device, an external support specifically designed to prevent SVG stretching after CABG.

Overall, the majority of the included RCTs demonstrated a reduction in intimal hyperplasia area and intimal-medial thickness (so-called atherosclerosis-predicting factors) in VEST-stented SVGs compared to non-stented controls.

This has been confirmed via a meta-analysis of RCTs, which included VEST I, VEST III, and US VEST, wherein the mean differences for intimal hyperplasia area and intimal-medial thickness, respectively, were −0.77 mm^2^ (95% CI: −1.10 to −0.45; I^2^ = 0%) and −0.06 mm (95% CI: −0.08 to −0.04; I^2^ = 0%) in favor of the VEST group. Despite this reduction, no difference in graft occlusion was seen between VEST-supported and unsupported SVGs (odds ratio (OR): 1.22; 95% CI: 0.88–1.71; I^2^ = 0%). Furthermore, no clinical outcomes were analyzed [14].

In another study-level meta-analysis of RCTs including only VEST III and US VEST (VEST I and its long-term follow-up VEST IV were excluded because of a variation in the device implantation technique in 30% of the patients), the results were similar. Interestingly, there was no difference in repeat revascularization (OR: 0.66; 95% CI: 0.27–1.64) between stented and non-stented SVGs at a mean follow-up of 1.5 years after CABG [15]. This is a key finding that outlines how patients receiving VEST-stented SVGs do not currently have an early post-operative clinical advantage compared to the standard of care (non-stented SVGs).

The importance of using meaningful clinical outcomes such as repeat revascularization or MACCE over surrogate outcomes (graft occlusion, intimal hyperplasia, intimal-medial thickness, and Fitzgibbon patency classification, among others) is paramount to estimating the impact of the VEST device at mid- and long-term follow-ups because the role of such surrogate endpoints is still unclear [16]. In this respect, a large, multi-center registry (VEST EU), including patients with at least one VEST-supported and one unsupported SVG, is currently collecting data on ischemia-driven revascularization (primary outcome) and may potentially add new, albeit non-randomized, information [17].

As we await the results of the extended follow-up in the cohorts enrolled in US VEST and the VEST EU registry, better long-term patency and clinical outcomes may safely be achieved using arterial conduits (radial artery, right internal thoracic artery), when clinically indicated [18,19,20], or the no-touch harvesting technique for SVGs [21].

In this regard, a post hoc analysis of VEST III has explored the effect of the harvesting technique on SVG patency and disease progression after CABG [22]. In this study, Sandner et al. compared patients undergoing open and endoscopic harvesting. The results showed that the open technique improved overall SVG patency after 2 years (90.8% vs. 73.9%; *p* = 0.01), particularly in stented SVGs (90.8% vs. 69.0%). In the open-harvest group, external stenting was also associated with reduced intimal hyperplasia area (−19.5%; *p* < 0.001)—to a greater extent than in the endoscopic harvesting group (−12.7%; *p* = 0.01)—and intimal-medial thickness (−25.0%; *p* < 0.001), as well as with improved Fitzgibbon patency class I, II, and III rates (OR: 2.84; *p* = 0.01). The fact that 72.2% of the stented SVGs displayed perfect patency after 2 years is a clinically relevant finding because lumen irregularities have been previously correlated with subsequent SVG failure [23,24].

It must be noted that in VEST III, the majority of SVG occlusions occurred in the first 6 months after CABG. This highlights once again the biomechanical properties of the VEST device, which is designed to mitigate disease progression rather than preventing early SVG failure.

Interestingly, to reduce the occurrence of early graft failure and, in turn, post-operative infarction, some authors have suggested using a simple valvulotomy of stented SVGs [25]. It is well-known that venous valves in arterialized SVGs may cause turbulence and dilatation of the vein segment nearer to the valve [26]. When an external stent is deployed, valve leaflet coaptation height and trans-valvular gradients may increase, leading to higher interference of graft flow patterns and accelerated early graft failure. A valvulotomy would therefore allow for a more uniform flow, avoiding thrombus formation in the valve recesses.

To conclude, as with other medical technologies, the evolution of the VEST device depends on a collaborative effort involving both research and surgeons’ expertise. This synergy will lead to device optimization and, hopefully, to improved short- and long-term clinical outcomes. While many years may pass before these goals are met, our duty is to offer the most effective means of treatment currently available.

## 5. Conclusions

The available randomized evidence suggests that a reduction in intimal hyperplasia can be achieved by using the VEST device. However, uncertainty remains over the long-term effects of VEST in reducing SVG failure and improving clinical outcomes.

## Figures and Tables

**Table 1:** Main characteristics and findings of the VEST studies.

	VEST I	VEST II	VEST III	VEST IV	US VEST
Author, year	Taggart, 2015	Taggart, 2016	Taggart, 2022	Taggart, 2018	Goldstein, 2022
RCT	Yes	No	Yes	Yes	Yes
Multi-center	Yes	No	Yes	Yes	Yes
Number of patients	30	30	184	30	224
Multi-vessel coronaropathy	Yes	Yes	Yes	Yes	Yes
Follow-up (months)	12	3–6	24	54	12
Patency VEST-SVGs (%)	70.0	86.2	78.3	70.0	76.7
Patency control-SVGs (%)	82.1	100	82.2	77.0	77.8
VEST reduced IH	Yes	-	Yes	Yes	No
VEST reduced IMT	No	-	Yes	Yes	Yes

IH: intimal hyperplasia; IMT: intimal-medial thickness; RCT: randomized controlled trial; SVG: saphenous vein graft/s; VEST: venous external support trial.

## Data Availability

Data and materials supporting the results of this analysis will be available from the corresponding author upon reasonable request.
